# Urinary podocyte microparticles are associated with disease activity and renal injury in systemic lupus erythematosus

**DOI:** 10.1186/s12882-019-1482-z

**Published:** 2019-08-05

**Authors:** Jian Lu, Ze Bo Hu, Pei Pei Chen, Chen Chen Lu, Jia Xiu Zhang, Xue Qi Li, Ben Yin Yuan, Si Jia Huang, Kun Ling Ma

**Affiliations:** Institute of Nephrology, Zhongda Hospital, School of Medicine, Southeast University, NO. 87, Ding Jia Qiao Road, Nang Jing City, 210009 Jiang Su Province China

**Keywords:** Podocyte-derived microparticles, Podocyte injury, Systemic lupus erythematosus

## Abstract

**Background:**

New non-invasive biomarkers are demanded to identify renal damage in various autoimmune-associated kidney diseases. Glomerular podocyte damage mediated by systemic lupus erythematosus (SLE) plays an important role in the pathogenesis and progression of lupus nephritis (LN). This study evaluated whether the podocyte-derived microparticles (MPs) were novel biomarkers of clinical and histological features in SLE patients with LN.

**Methods:**

A cross-sectional study, including 34 SLE patients and 16 healthy controls, was designed. Urinary annexin V^+^ podocalyxin^+^ MPs of all participants were quantified by flow cytometry. The correlation of podocyte-derived MPs with clinical and histological parameters of SLE patients was analysed.

**Results:**

The number of annexin V^+^ podocalyxin^+^ MPs from urine samples were markly increased in patients with SLE. Furthermore, the level of urinary podocyte-derived MPs was positively correlated with the SLE Disease Activity Index (SLEDAI) score, anti-dsDNA antibody titre, erythrocyte sedimentation rate, and proteinuria. Conversely, it was negatively correlated with the level of complement C3 and serum albumin. The number of urinary podocyte-derived MPs was significantly increased in SLE patients with high activity indices. Receiver operating characteristic (ROC) curves were calculated to assess the power for podocyte-derived MP levels in differentiating between SLE patients with and without LN. Podocyte-derived MP levels were able to differentiate between SLE patients with mild disease activity, as well as those with moderate and above disease activity. SLE patients showed increased podocyte-derived MP excretion into the urine.

**Conclusions:**

These findings suggest that the change in urinary podocyte-derived MP levels could be useful for evaluating and monitoring SLE disease activity.

## Background

Systemic lupus erythematosus (SLE) is a systemic autoimmune disease with multiple organ injuries. Lupus nephritis (LN) is recognized as one of the most severe organ manifestations of SLE and is characterized by proteinuria, haematuria and progressive renal dysfunction. The pathogenesis of LN involves the glomerular deposition of autoantibodies related to self-antigens, the activation of complement system, the infiltration of inflammatory cells, and the over-production of proinflammatory cytokines and chemokines. At present clarifying the pathogenesis of LN, monitoring the degree of disease activity and taking effective measures to slow the progression of LN are substantial challenges.

Microparticles (MPs), a subtype of extracellular vesicles (EVs), are released by outward blebbing of the plasma membrane after the externalization of phosphatidylserine (PS) during cell activation and apoptosis [1]. The MPs carry various bioactive molecules and thereby serve as vectors for intercellular interaction. Interestingly, MPs can display biological activities associated with thrombosis, inflammation, and immune response [2]. Thus, MPs may be involved in the pathological and physiological processes of some diseases. Recent studies found that podocyte-derived MPs played crucial roles in the pathogenesis of some glomerular and non-glomerular diseases, being a novel early biomarker reflecting the damage of glomerular cells [3]. Gilani et al. [4] reported that renal injury in preeclampsia was associated with an elevated urinary podocin+ EV-to-nephrin+ EV ratio. Compared with patients with essential hypertension and healthy volunteers, renovascular hypertensive patients had elevated urinary podocyte EV levels. Podocyte EVs may reflect primary kidney damage-related podocyte injury [5].

The detection and analysis of urinary podocyte-derived MPs could be a potential non-invasive method for the monitoring of the progression of glomerular diseases. Therefore, this study aimed to identify podocyte phenotype, detect podocyte-derived MP level in the urine of SLE patients with and without LN and examine this association with clinical parameters.

## Methods

### Study design of all subjects

Total 34 patients with SLE (4 men and 30 women) and 16 healthy controls (HCs, 10 men and 6 women) were enrolled. All the SLE patients were diagnosed according to American College of Rheumatology Classification Criteria, and the SLE Disease Activity Index (SLEDAI) was used to assess the disease activity of SLE [6]. The LN (*n* = 19) was defined as impaired kidney function with proteinuria (> 0.5 g/24 h proteinuria) or non-infective leukocyturia, haematuria or biopsy-proved active glomerulonephritis. Fifteen SLE patients without LN were also included. Baseline data including serum creatinine (Cr), 24-h proteinuria, complement (C3 and C4) levels and anti-double strand DNA (anti-dsDNA) antibody were measured. Estimated glomerular filtration rate (eGFR) was estimated by the MDRD formula. All subjects signed the written informed consent. The study was approved by the ethics committee of Zhongda hospital, Southeast University.

### Renal histopathology

Twelve biopsy-proven LN patients were recruited in this study. Histopathological assessment of renal biopsy specimens was performed with light microscopy, immunofluorescent staining, and electron microscopy. The pathological classification of LN was based on the histopathological assessment according to the ISN/RPS 2003 Classification standard [7]. The active and chronic indices of pathological damage were accessed by semi-quantitative scoring of pathological features.

### Isolation of microparticles from spot urine among all participants

Fifty ml of morning spot urine was collected in a sterile bottle from each patient and healthy control. After collection, protease inhibitor cocktail was added according to the manufacturer’s instructions [8]. Debris and cells were then removed by centrifugation (3000 g for 10 min). The supernatant was transferred to a new tube, and then centrifuged (18000 g for 20 min) to pellet MPs. The MPs were resuspended in 300 μl of annexin V Binding Buffer (BD Biosciences, US), and then MP suspension was immune-labelled with specific antibody and analysed by flow cytometry. For MP labelling, an allophycocyanin-labelled annexin V antibody (BD PMG, China) was used for identifying events as total MPs. Among total MPs, a phycoerythrin-conjugated Podocalyxin antibody (Ebioscience, USA) was employed to identify podocyte origin.

### Statistical analysis

Data analysis was performed with SPSS (Version 20.0). Normally distributed data was analysed with the Student’s *t*-test and χ2 test. The Kruskal-Wallis with post hoc analyses were performed for non-normally distributed data. The association between podocyte MP levels with clinical parameters was analysed with Spearman’s correlation test. *P* < 0.05 was considered as significant difference.

## Results

### Baseline clinical characteristics of all subjects

The baseline characteristics of all subjects were summarized in Table 1. Of all 34 SLE patients, 19 SLE patients had active LN, and the other 15 SLE patients had only SLE without LN.

**Table 1:** Demographic and clinical data of SLE patients and healthy volunteers

Variable	SLE patients (*n* = 34)	Controls (*n* = 16)
Sex (female/male) (n)	30/4 *	6/10
Age (years)	48.2 ± 12.7 *	36.2 ± 8.0
Disease duration (years)	4 (0.25, 9)	ND
SLEDAI score	8 (5.5, 15.5)	ND
Anti-ANA positive	34	ND
Anti-dsDNA (titre U/ml)	10 (73, 588.4)	ND
C3 (mg/dl)	0.71 ± 0.34	ND
C4 (mg/dl)	0.15 ± 0.89	ND
Serum creatinine (μmol/L)	70 (62.75, 86.0)	71 (84, 89)
eGFR (mL/min/1.73 m^2^)	104.05 (82.43, 119.23)	96.30 (83.09, 101.68)
Proteinuria (g/24 h)	0.58 (0.08, 3.15)	ND
Hematuria	28/6	ND
Cellular casts	24/10	ND

The data are expressed as the mean ± SD or median (IQR). *SLEDAI* SLE detection assessment index, *Anti-dsDNA* anti-double-stranded DNA, *C3* complement 3, *C4* complement 4, *eGFR* estimated glomerular filtration rate, *ND* not determined, Differences between the two groups were analysed by χ2 or Mann-Whitney U tests, respectively. *P < 0.05 compared with controls

### Compared with healthy controls, SLE patients had elevated urinary podocyte-derived MPs

**Fig. 1:**
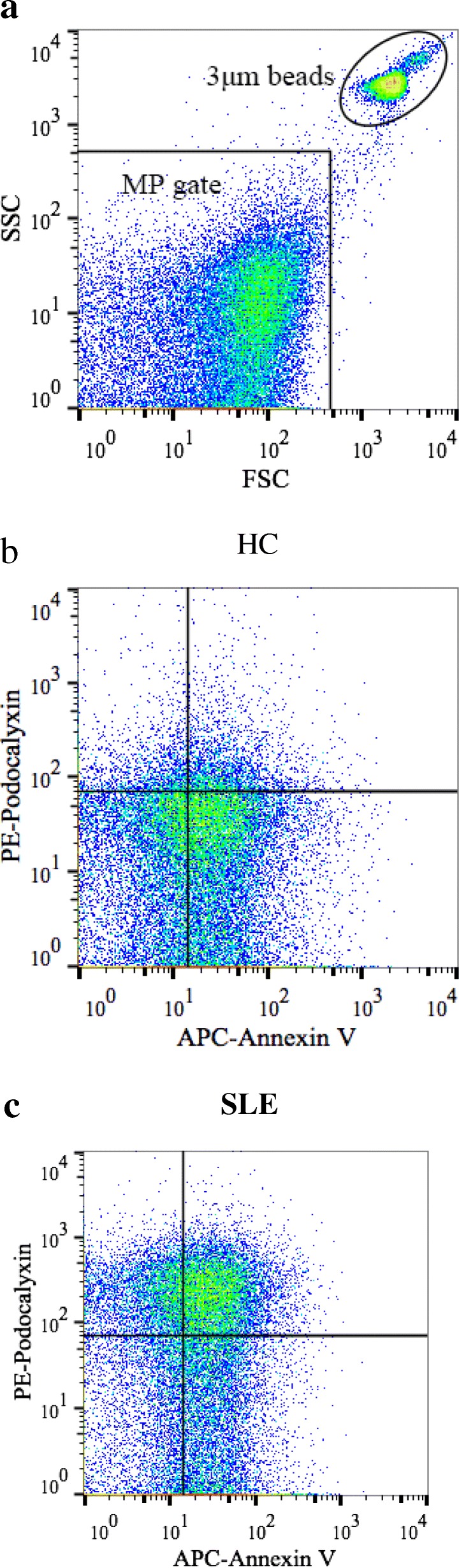
Flow cytometry analysis strategy of the number of podocyte-derived microparticles (MPs) in all enrolled participants. Urinary MPs were labelled with Annexin V and podocalyxin (mAb), and analysed with a FACS Calibur flow cytometer. **a** The size of particles to be analysed was defined with certain gate setting strategy. **b**-**c** Representative images of dectation of podocyte-derived MPs isolated from the urine. Absolute numbers of the urinary podocyte-derived MPs were determined as particles with annexin V and podocalyxin double positive (Counts × 10^6^/ml urine)

**Fig. 2:**
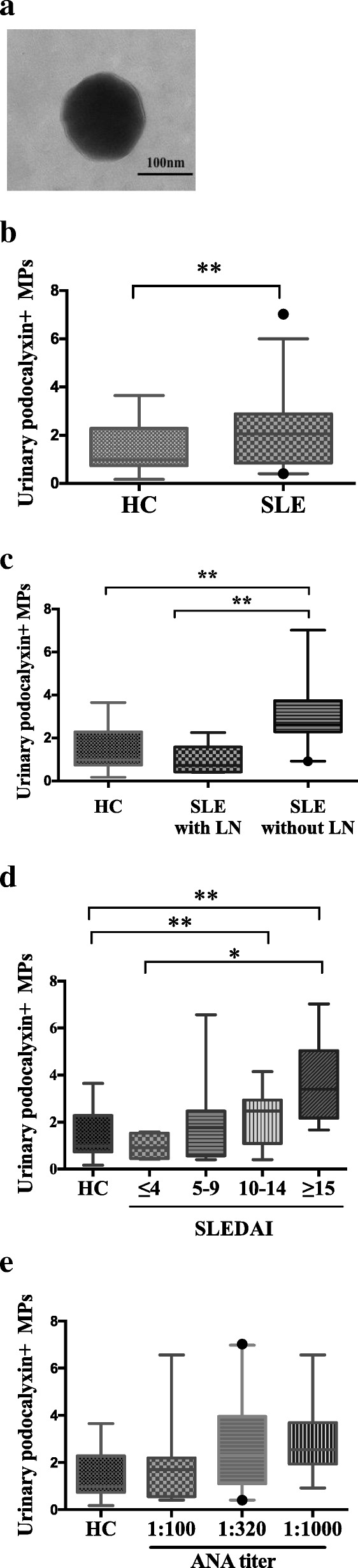
Comparison of the levels of urinary podocyte-derived MPs from SLE patients group and healthy control group. **a** Urinary MPs from a SLE patient were observed by transmission electron microscopy (× 40000). **b** The absolute numbers of podocyte-derived MPs in two groups. **c** Analysis of urinary podocyte-derived MPs in two subgroups of SLE patients (SLE without LN and SLE with LN) and healthy controls. **d** Analysis of podocyte and MPs in patients with SLE for different disease activity (defined by SLEDAI score) and healthy control. (**e**) Analysis of podocyte and MPs in patients with SLE for different ANA titer and healthy control. Data are shown as boxes displaying median with interquartile range. HC, healthy control; LN, lupus nephritis. *P < 0.05; **P < 0.01

As shown in Fig. 1, urinary podocyte-derived MPs were detected by flow cytometry in healthy controls and SLE patients. Figure 1a showed representative methods for MP detection by FACS Calibur flow cytometry. Transmission electron microscopy observation showed that there were round MPs in the urine of SLE patients (Fig. 2a). Compared with healthy controls, there was a significant increase of podocyte-derived MPs levels (podocalyxin and annexin-V double positive) in SLE patients (Fig. 2b). Moreover, the level of urinary podocyte-derived MPs in patients with LN was higher than that in patients without LN (Fig. 2c). The change of urinary podocyte-derived MPs was compared among these patients according to the SLEDAI score. The results demonstrated that as SLEDAI scores increased, the number of urinary podocyte-derived MPs significantly increased (Fig. 2d).

### Association analysis of urinary podocyte-derived MPs with clinical indexes in SLE patients with LN

**Fig. 3:**
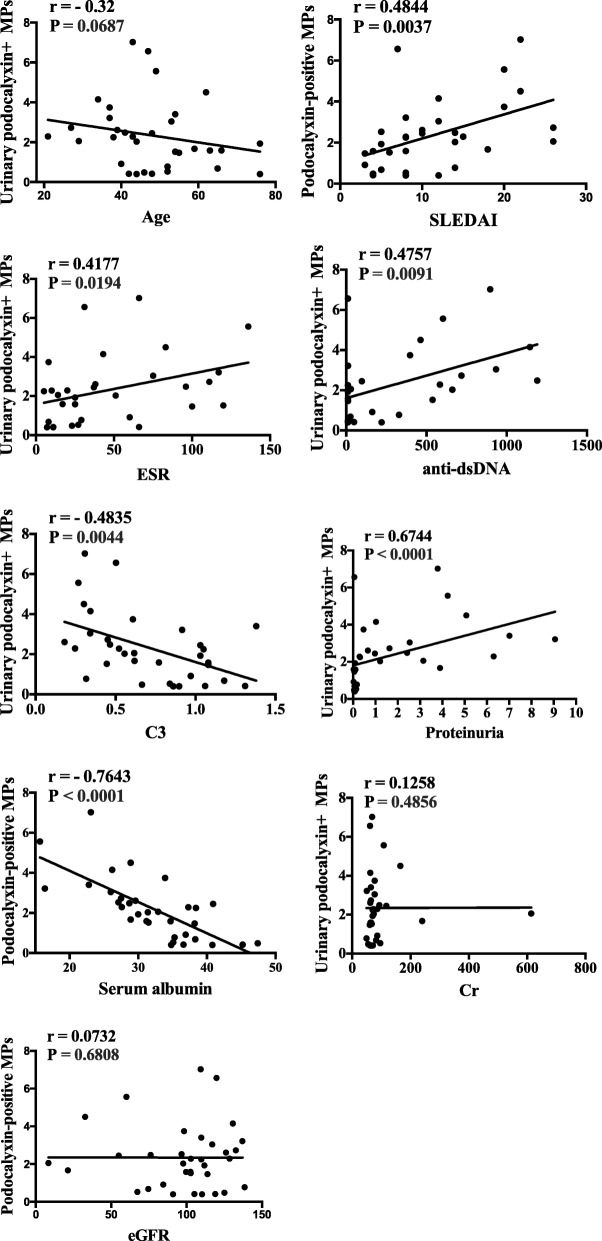
Correlation analysis between levels of urinary podocyte-derived MPs and disease activity in SLE patients. The correlation was assessed between the levels of the kind of MPs and some clinical indexes. The correlation coefficient from Spearman’s correlation test and *P*-values were indicated

To determine whether urinary podocyte-derived MP levels were associated with renal activity, we performed a correlation analysis between various clinical parameters and urinary podocyte-derived MP levels. As shown in Fig. 3, levels of urinary podocyte-derived MPs were positively correlated with higher SLEDAI scores, higher anti-dsDNA antibody titres, higher ESR, and increased proteinuria; conversely, they were negatively correlated with complement C3 and serum albumin level. However, there was no significant association between the levels of urinary podocyte-derived MPs and eGFR or serum creatinine levels.

### The comparison analysis of urinary podocyte-derived MPs with histological features in SLE patients with LN

**Fig. 4:**
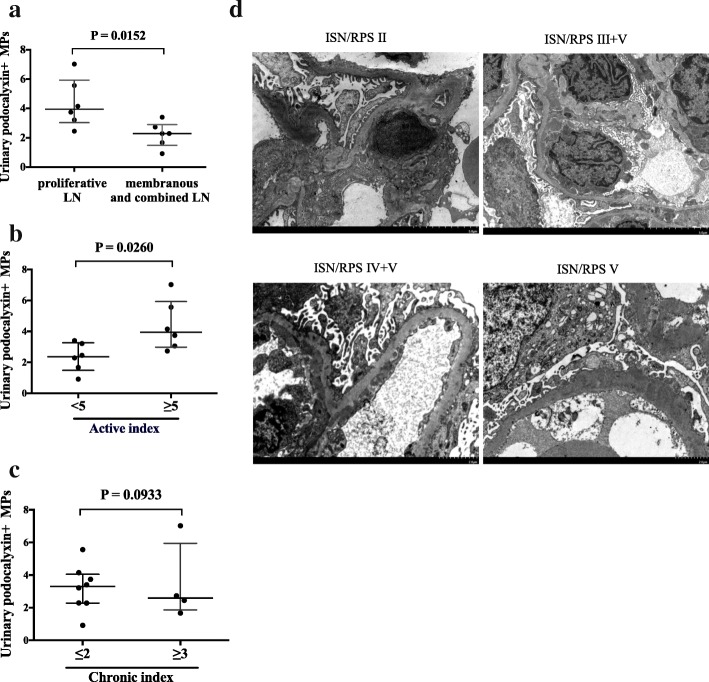
The comparison analysis of urinary podocyte-derived MPs with histological features in SLE patients with LN. **a** Analysis of urinary podocyte-derived MPs in two subgroups of biopsy-proven LN patients: proliferative LN (pure Type II, Type III, and Type IV), Type V LN, and combined LN (Type III + V and Type IV + V). **b** Analysis of urinary podocyte-derived MPs in biopsy-proven LN patients with different degrees of activity indices. **c** Analysis of urinary podocyte-derived MPs in biopsy-proven LN patients with different degrees of chronicity indices. Data are shown as scatter dot plot displaying median, interquartile range. *P < 0.05. **d** The effacement degree of podocyte foot processes in pure proliferative LN, membranous LN, and combined membranous LN observed by electron microscopy

Among 19 SLE patients with active LN, 12 patients underwent kidney biopsies. The pathological classification was done according to the ISN/RPS 2003 classification system. Since the degree of podocyte injury could be different in different pathological types of LN, we further divided the biopsy-proven LN patients into two groups: proliferative LN (pure Type II, Type III and Type IV), Type V LN and combined LN (Type III + V and Type IV + V). As shown in Fig. 4a, the significant increase levels of podocyte-derived MPs were observed in the SLE patients with proliferative LN compared to those patients with Type V LN and combined LN. This was in concordance with that the higher number of urinary podocyte-derived MPs was in LN patients with higher activity indices. Whereas the change of urinary podocyte-derived MPs was no difference among LN patients with increased chronic indices. These results indicated that in active LN, there were more podocyte-derived MPs shed into urine. In addition, we observed the ultrastructure change of podocyte foot processes by electron microscopy. We found that there were a variety of morphological podocyte lesions among different pathological types of LN. In proliferative LN with Type II, the podocyte foot process was fused slightly. However, there were extensive effaced podocyte foot processes in LN with Type III/V, Type IV/V, and Type V. These findings suggest that the urinary levels of podocyte-derived microparticles are closely correlated with the active indices and the ultrastructure change of podocytes.

### Performance of urinary podocyte-derived MPs in differentiating disease activity of SLE patients with or without LN

**Fig. 5:**
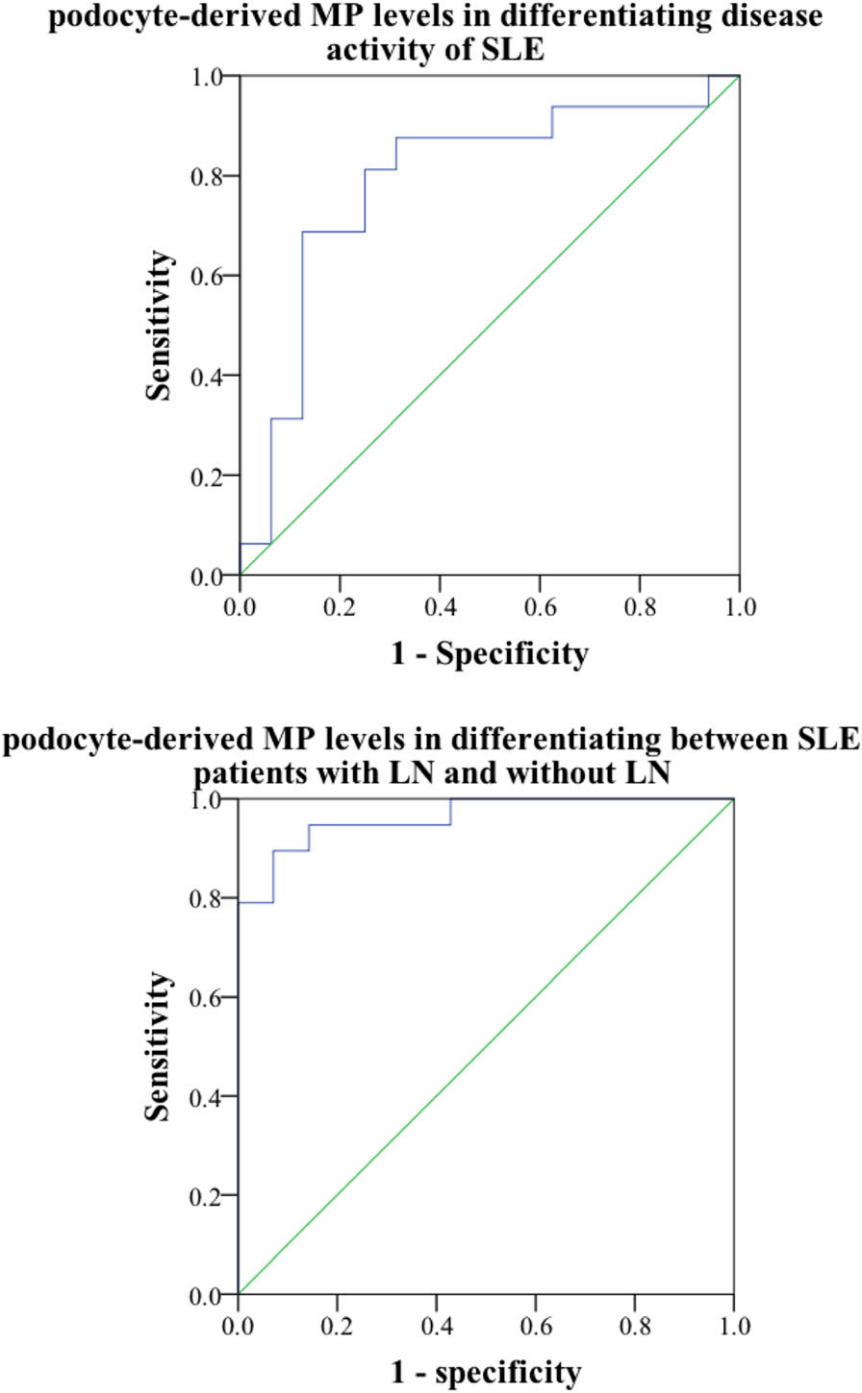
ROC curves were calculated to assess the power for podocyte-derived MP levels in differentiating between SLE patients with LN and without LN [AUC = 0.962 (95% CI 0.905–1). Additionally, podocyte-derived MP levels were able to differentiate between SLE patients with mild disease activity (SLEDAI score < 4), and moderate and above disease activity (SLEDAI score ≥ 5) (AUC = 0.789, 95% CI 0.62–0.958)

We further performed receiver operating characteristic (ROC) curve analysis. As displayed in Fig. 5, urinary podocyte-derived MPs were used in differentiating SLE in the active LN group from the without LN group (AUC; 0.962 (95% CI 0.905–1)). The area under the ROC curve for urinary podocyte-derived MPs in the diagnosis of LN disease activity was 0.789 (95% CI 0.62–0.958).

## Discussion

Podocytes are critical cell components of the glomerular filtration barrier, which belong to highly differentiated cells and are almost non-renewable. Podocytes play key roles in maintaining the selective filtration of the glomerulus. Once podocyte injury occurs, it results in worsening proteinuria and renal failure. In LN, a major complication of SLE is often manifested with mesangial cell proliferation, decreased podocyte density, foot process fusion, and the change of podocyte phenotype. More and more studies have focused on podocyte damage in LN [9, 10]. Specifically, podocyte lesions in LN are explained by the following patterns: one is focal and diffuse proliferative LN injuries, concerning the immune complex deposition with the structural podocyte damage; the other is non-proliferative LN, presenting with diffuse podocyte structural foot process effacement and dysfunction [11]. In both forms of LN, the diffused podocyte foot process effacement and reduced expression of podocyte-specific proteins [12], along with an increased excretion of podocyte-related products in the urine [10], are powerful evidence of podocyte, as a victim not to be ignored.

A main finding in this study was that urinary podocyte-derived MPs were increased in SLE patients compared with those in healthy controls, especially in those with active renal injuries characterized by proteinuria and higher SLEDAI scores. MPs are released from apoptotic or activated cells in response to various stimuli [13]. Urinary MPs, as a subtype of EVs, are enriched in functional cargo proteins and released from the different epithelial cells of the whole urinary tract. The cellular sources of urinary MPs can provide important information about disease pathogenesis. It is increasingly clear that EVs are present in urine and may be of significant clinical use. Subsequent studies have reported that the level of EVs in the urine is elevated in patients with glomerular diseases [14] such as focal segmental glomerulosclerosis [15], minimal change disease and diabetic nephropathy [16]. Moreover, the pathogenic roles of circulating MPs derived from various cellular sources, such as platelet-derived MPs in rheumatoid arthritis [14] and SLE patients [17], have been demonstrated. However, to date, there are only a few studies to reveal urinary MPs as new urine biomarkers [18, 19]. A increase of numbers of MPs observed in SLE patients most likely result from abnormal cell activation and apoptosis together with defective clearance of MPs, which are all mechanisms related to underlying SLE pathogenesis, as discussed elsewhere [20]. Healthy controls had certain levels of urinary MPs positive for podocalyxin. Podocalyxin is a transmembrane protein expressed on the apical cell membrane of glomerular podocytes [21]. Podocalyxin physically functions to maintain podocyte structure and the integrity of slit diaphragm. Upon damage, podocyte would experience various morphological changes, such as foot processes fusion, decrease of the cell density and the formation of pseudocysts, ultimately stripping from the glomerular basement membrane and falling off into the urine. It was reported that podocalyxin is shed from injured podocytes into the urine as small vesicles [22]. These results are in line with the idea that the shedding process of podocalyxin-related vesicles can be an indicator of the state of the podocyte in situ. Disease severity was identified by the SLEDAI score, and a SLEDAI score > 10 was a marker of highly active disease [23]. SLEDAI has been reported to be associated with the presence of active LN [24].

Our results, for the first time, demonstrated that the level of urinary podocyte-derived MPs was closely correlated to SLE disease activity, which was mainly shown by the strong positive association between the number of podocyte-derived MPs in the urine and the titre of anti-dsDNA antibodies. Serum levels of anti-dsDNA antibodies and Complement 3 are two important diagnostic indexes of SLE and play key roles in the pathogenesis of LN. Anti-dsDNA antibodies bind with circulating DNA and then form circulating immune complexes, thus resulting in renal damage. According to the report, anti-dsDNA antibodies can directly have cross reactions with the podocyte proteins such as α-actinin 4, inducing podocyte cytoskeleton reorganization and fused podocyte morphology, eventually resulting in LN [25]. Quantitative detection of anti-dsDNA antibodies can evaluate the disease activity of SLE. The complement system is intimately related to SLE. Low serum level of Complement 3, as a biomarker of active SLE, may reflect complement activation [26]. Although the sensitivity and specificity of Complement 3 (75%/71%) for identifying the degree of renal injuries is relatively low, there are certain implied values of this positive correlation for clinical decisions. The measurement of 24-h proteinuria in urine samples is the classical biomarker for assessing LN, and it somehow reflects eventual renal outcome. Proteinuria is partly consistent with histological index activity changes in LN, and a significant increase in urinary podocyte-derived MP levels with the gravity of histological features was observed. Serum creatinine (Cr) and eGFR are standardized tools for estimating renal function [27]. No possible association between podocyte-derived MPs and Cr and eGFR was acceptable. Vesicle shedding has been regarded as an early sign of podocyte injury. However, the patients we enrolled were not conditioned in the early stage of CKD.

Regardless of the different causes for podocyte injury, the characteristic morphological change of podocyte is the foot processes effacement. The cytoskeleton change of podocyte is often accompanied by the release of podocyte-derived MPs. Our results showed that the manifestations of foot process fusion varied among the pathological phenotypes of LN, especially for the combined membranous patterns (Type III + V or IV + V). There was more podocyte-derived MPs release in the pathological active LN groups. While the levels of podocyte-derived MPs were no difference among LN patients with increased chronic indices. It meaned that as the podocyte foot process fusion is aggravating from the acute to chronic damage, the number of podocytes in situ was reduced and the corresponding shedding of podocyte-derived MPs was also decreased.

ROC analysis was performed to calculate the area under the curve (AUC) for evaluating the diagnostic performance of the models. Our data demonstrated that the AUC of podocyte-derived MPs was 0.789, when SLEDAI score ≥ 5 was designated as light or moderate clinical disease activity of LN. The AUC of podocyte-derived MPs was 0.962, when proteinuria (> 0.5 g/24 h) was designated a diagnostic LN pathological activity indicator. In summary, podocyte-derived MPs can be a reliable indicator that reflect the disease activity of SLE.

There are an increasing number of urine proteins, including chemokines, cytokines, growth factors and adhesion molecules [26]. These proteins have been evaluated as candidates for SLE biomarkers, but few have been validated and implied for clinical practice. EVs are not only the witnesses, but the contributors to the diseases. The biological information of the particles and the effect of the downstream target cells, especially tubular epithelial cells, need to be further discussed.

The limitations of this study include the following aspects. First, the small sample size may affect the reliability of the results and further analysis. Thus, a large sample and prospective follow-up studies are also needed. Second, this study could not link higher podocyte-derived MP counts to the progression of kidney disease, which would require longitudinal studies.

## Conclusion

To summarize, our studies firstly demonstrated that the levels of urinary podocyte-derived MPs were dramatically increased in patients with SLE. Moreover, the levels of urinary podocyte-derived MPs were positively associated with SLE disease activities. Further studies will be required to determine the release mechanisms of podocyte-derived MPs and to explore whether the MPs released from glomerular cells may be used as novel biomarkers for the early prediction and monitoring of disease activity in SLE.

## Data Availability

Not applicable.

## Abbreviations

anti-dsDNA: anti-double strand DNA
C3: complement 3
Cr: serum creatinine
eGFR: estimated glomerular filtration rate
EVs: Extracellular vesicles
LN: Lupus nephritis
MPs: microparticles
PS: Phosphatidylserine
ROC: Receiver operating characteristic
SLE: Systemic lupus erythematosus
SLEDAI: SLE Disease activity index
